# Does It Pay?

**Published:** 1899-12

**Authors:** 


### Does It Pay?

In the course of his professional life many a man must pause and ask himself this question. In the Cincinnati *Lancet-Clinic* of September 9, there are two communications, one entitled "Does the Practice of Medicine Pay?" in which the writer, Dr. Monroe of Louisville, takes a rather pessimistic view, dwells upon the many hardships of a doctor's life, and dismisses the subject with these words: "Therefore, taking everything into consideration, I think I am perfectly safe in saying, in closing this paper, that it does not pay, financially, to be a physician."

The second paper had for its caption, "Does It Pay to Live?" in which the intellectual pleasures constituting

"The soul's calm sunshine and the heartfelt joy"

are dwelt upon, and it is pointed out that there are other objects in life beside and beyond those associated with finance.

This writer, Dr. Woodruff, believes that no greater reward can come to a physician than the ability to look back upon time well spent and the consciousness of good example stamped upon the community. He says: "The opportunities of relieving suffering and inspiring hope in the despondent are so numerous in the daily rounds of a doctor, that any physician who has spent his life in the conscientious discharge of his duties, may answer the question in the affirmative."

But, after all, it is the individual point of view. "As a man thinketh in his heart, so is he."—*New York Medical Record.*

